# Prediction of IL4 Inducing Peptides

**DOI:** 10.1155/2013/263952

**Published:** 2013-12-30

**Authors:** Sandeep Kumar Dhanda, Sudheer Gupta, Pooja Vir, G. P. S. Raghava

**Affiliations:** Bioinformatics Centre, CSIR-Institute of Microbial Technology, Chandigarh 160036, India

## Abstract

The secretion of Interleukin-4 (IL4) is the characteristic of T-helper 2 responses. IL4 is a cytokine produced by CD4+ T cells in response to helminthes and other extracellular parasites. It has a critical role in guiding antibody class switching, hematopoiesis and inflammation, and the development of appropriate effector T-cell responses. In this study, it is the first time an attempt has been made to understand whether it is possible to predict IL4 inducing peptides. The data set used in this study comprises 904 experimentally validated IL4 inducing and 742 noninducing MHC class II binders. Our analysis revealed that certain types of residues are preferred at certain positions in IL4 inducing peptides. It was also observed that IL4 inducing and noninducing epitopes differ in compositional and motif pattern. Based on our analysis we developed classification models where the hybrid method of amino acid pairs and motif information performed the best with maximum accuracy of 75.76% and MCC of 0.51. These results indicate that it is possible to predict IL4 inducing peptides with reasonable precession. These models would be useful in designing the peptides that may induce desired Th2 response.

## 1. Introduction

Cellular immune response to a pathogen is mediated through the processing and presentation of antigen on the surface via major histocompatibility complex (MHC). The exogenous antigens are processed through lysosome and presented by MHC class II. The loaded peptide on MHC class II interacts with CD4+ T cells and a pattern of cytokine is synthesized and secreted. Depending upon the cytokines secreted, the T-helper cells polarize into diverse T-cell populations like Th1, Th2, Th17, or iTregs [1]. In Th2 cell population, interleukin-4 is the major cytokine secreted. IL4 had been shown to play a critical role in diverse biological activities. This cytokine promotes the proliferation and differentiation of antigen presenting cells [2]. IL4 also plays a pivotal role in antibody isotype switching and stimulates the production of IgE. This cytokine has been applied in the treatment of autoimmune disorder like multiple myeloma [3], cancer [4], psoriasis [5], and arthritis [6]. IL4 has also been extensively applied to inhibit detrimental effect of Th1 [7]. Hence, for the rational development of better immunotherapy/vaccines to provide protection against infection, it is pivotal to assess the immune response generated by these antigens.

Although this identification demands experimentally validating the immune response generated by each antigen, this investigation is time-consuming, cumbersome, and expensive task since the possible antigens and corresponding fragments range in millions [8–11]. Thus, the initial screening of whole of pathogen's proteome for potential antigens/regions demands systematic computational approach. Over the last decade, tremendous efforts has been made for identifying antigenic regions or epitopes within antigens that can activate desired arm of immune responses against a number of pathogens. This has resulted into development of a number of software applications, databases, and webservers that assist researchers to design and select antigens to activate various arms of the host immune system like humoral, cellular, and innate immunity. In order to facilitate users, in the past a number of methods have been developed to predict antigenic regions/peptides or different types of epitopes such as MHC class I/II binders, TAP binders, linear/conformational B-cell epitopes, and pathogen associated molecular patterns [12–15].

In the past, several methods have been developed for identification of MHC class II binders that may activate T-helper cells. These T-helper cells induce different types of cytokines like IL-4 and IFN-gamma. Presently, available methods provide no information about the type of cytokine a MHC II binding peptide will induce. In order to address this issue, an attempt has been made to develop a method for predicting IL-4 inducing MHC II binding peptides.

## 2. Methodology

### 2.1. Datasets Source and Processing

#### 2.1.1. Main Dataset

It is important to select the right dataset for developing a prediction method. The performance of the method is largely dependent on the datasets used for training a model. In this study datasets were generated from publicly available immune epitope database (IEDB) [16]. We extracted experimentally validated MHC class II binding T-helper epitopes; peptides having length shorter than 8 residues and longer than 22 residues were removed. Finally, we got unique 904 IL4 inducing and 742 noninducing MHC class II peptide sequences; we called these peptide sets as positive and negative sets, respectively. This dataset was created without any restrictions of host and source of epitopes.

#### 2.1.2. Alternative Dataset

Since the main dataset includes only MHC class-II binders, the prediction algorithm (based on the above dataset) can only predict IL4 inducing peptides from MHC class-II binders. We are also interested in discriminating the IL4 inducing peptide from the random peptides. Thus, we created an alternate dataset for building prediction models that can be used for mapping IL4-inducing peptides in antigens. Our alternate dataset has random peptides as negative set instead of non-IL4-inducing peptides. We generated IL4 noninducing (negative examples), from SwissProt proteins.

### 2.2. Peptide Length and Amino Acid Position Analysis

We first analyzed the IL4 inducing positive and noninducing negative sequences, to comprehend the preferred peptide length for both positive and negative peptides, by using R-package for creating boxplot [17]. We also tried to understand the preference of specific amino acids at a specific position. For this, we created a two-sample logo from first 15 amino acids from N-terminal of all the peptides, using the two-sample logo software [18].

### 2.3. Motif Analysis

The recognition of functional motifs in peptide or proteins constitutes an important element in functional annotation of sequences [19]. In the present study, we have employed publicly available software MERCI for selection of exclusive motifs in IL4 inducing and noninducing MHC class II binding peptides [20]. MERCI compares both the positive and negative input sequences and selects the specific motifs in the positive datasets. Thus, in our analysis, to understand specific motifs for both IL4 inducing and noninducing peptides, we analyzed our datasets using two-step strategy. In this approach, we first provided MERCI with IL4 inducing peptides as positive input and IL4 noninducing peptides as negative input and extracted the motifs for IL4 inducing peptides. In the next step, we reversed the datasets; that is, we provided MERCI with IL4 noninducing peptides as positive datasets and IL4 inducing peptides as negative datasets and obtained the motifs important for IL4 noninducing peptides.

We further explored 100 degenerate motifs using three kinds of classification: (i) none, (ii) Koolman-Rohm [21], and (iii) Betts-Russell [22]. Top 10 motifs were extracted based on their unique sequence coverage. These different classification methods were employed to further discover different motifs in positive and negative peptides. Finally, unique motif containing peptides from both IL4 inducing positive dataset and IL4 noninducing negative dataset were selected, to calculate overall motif coverage in these sequences.

### 2.4. Amino Acid and Dipeptide Compositions

We next analyzed the residue composition of these IL4 inducer and noninducer peptides. For this, we used in-house Perl scripts to calculate the amino acid composition of the peptide and summarize the intact epitope information in a fixed vector length. The algorithm calculates amino acid composition (AAC) using the following formula and a vector of dimension 20 is used to represent amino acid composition of a peptide:

(1) composition  of  amino  acid  (i)  =total  number  of  amino  acid(i)×100total  number  of  all  amino  acids  in  epitope,

where i can be any amino acid.

Likewise, the algorithm calculates dipeptide composition (DPC) and a vector of dimension 400, representing a peptide, using the following formula:

(2) composition  of  dipeptide  (i+1)  =total  number  of  dipeptide(i+1)×100total  number  of  all  possible  dipeptides  in  epitope,

where i can be any amino acid and (i + 1) is dipeptide pair with next residue in epitope.

### 2.5. Amino Acid Pairs

Amino acids pairs (AAP) based method represents the input epitope by a vector of fixed vector length (400) by incorporating the information from each amino acid pair and their propensity in the given dataset. This approach had shown its potential in past for predicting B-cell epitope [23].

### 2.6. Calculation of Binary Patterns

Here, we converted positive and negative examples into binary codes, where each amino acid is represented by a vector of dimension 20 (e.g. Ala by 1,0,0,0,0,0,0,0,0,0,0,0,0,0,0,0,0,0,0,0; Cys by 0,1,0,0,0,0,0,0,0,0,0,0,0,0,0,0,0,0,0,0). Using these binary vectors, different peptides were represented, such as a 15-amino acid long peptide which is represented by a vector of dimension 300 (15 × 20).

### 2.7. Support Vector Machine Learning Approach

In this study, classification models have been developed using machine learning technique support vector machine (SVM). In order to implement or develop SVM models, we used software SVMlight [24]. SVM models were developed using different features such as amino acid composition and amino acid pairs. In order to train or optimize the performance, we tuned all SVM parameters including three types of kernels (linear, polynomial, and radial bias).

### 2.8. Hybrid Approach

We further employed a hybrid approach, where we combined the predictions from both motif and model based methods. In a hybrid approach, the weight of +1 was given to the peptide having IL4 motif (exclusively found in IL4 inducing peptides) and −1 was given to peptide having a non-IL4-motif (exclusively found in non-IL4-inducing peptides). We developed several hybrid models depending on the type of vector inputs used for SVM based prediction.

### 2.9. Evaluating the Performance of Models Validation

In order to develop reliable prediction models, we trained and tested our models using fivefold cross-validation technique. In this analysis, the whole dataset is divided randomly into five equal parts, and each time four sets are used for training our models and remaining set is used for testing. This procedure is repeated five times so that each set is tested once and four times it is used for training. The final performance of the model is evaluated by averaging the performance of models on each set. The performance of models was measured using the following standard parameters, that is, sensitivity, specificity, accuracy, and Matthew's correlation coefficient (MCC).

### 2.10. Data Analysis

In order to understand the properties of the IL4 inducers, we analyzed both IL4 inducer (IL4+) and noninducer (IL4−) MHC class II binding epitopes extracted from IEDB. There are several studies where authors have exploited physiochemical properties (PCPs) of peptides to discriminate one class of peptide from other classes [25]. We examine various PCPs (such as hydrophilicity, hydrophobicity, charge, steric effect, side bulk, pI, hydropathy, and amphipathy) of IL4+ and IL4− peptides [26]. In our analysis, we calculated the average of PCP in three different manners: (i) Average of that PCP at a particular position of 15 N-terminal or C-terminal residues; (ii) average of the sum of PCP of all the peptides; for example, hydrophilicity of every peptide was calculated by the sum of hydrophilicity of every residue and average of all IL4+ peptides was taken; (iii) average of the sum of PCP of selected residues of N′ and C′ terminals, after analysis of discriminating residue positions; for example, 1, 2, 3, 5, and 12 positions of N′ terminal were selected for hydrophilicity (see Figure 1S in Supplementary Material avialable online at http://dx.doi.org/10.1155/2013/263952).

## 3. Results

### 3.1. Peptide Length Analysis

**Figure 1:**
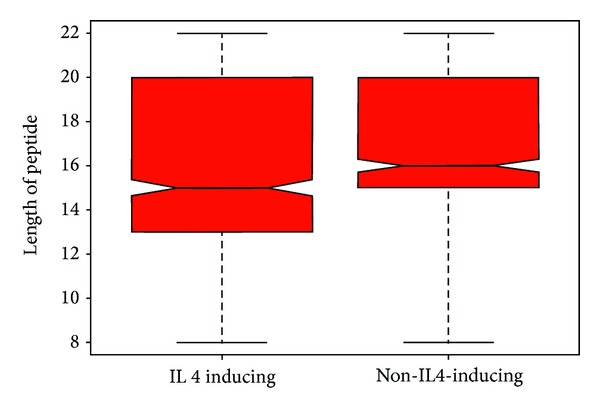
Box plot to represent the variation of peptide length in IL4 inducing and non-IL4-inducing dataset.

We first compared the length of the two types of peptides and observed that average length of Il4 inducing and noninducing peptides is not significantly different. We could not find any significant relation between length of sequence and its potential to induce IL4 production (Figure 1).

### 3.2. Amino Acid Composition

**Figure 2:**
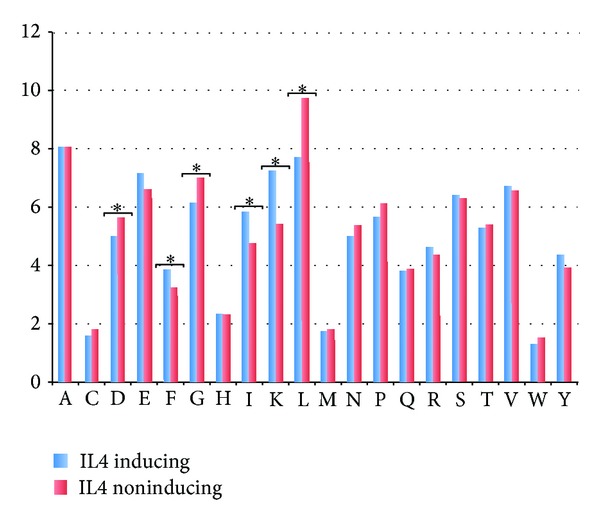
Bar plot representing the average percentage composition of residue in IL4 inducing and non-IL4-inducing datasets. ∗ here represents the significantly different residues at *P* value <0.05.

We also compared amino acid composition of IL4+ and IL4− peptides and found compositional biasness between two types of peptides. It was observed that residues E, F, K, and I are more abundant in IL4 inducing peptides, while IL4 noninducing sequences majorly include G, D, and L (Figure 2).

### 3.3. MHC Alleles Skewness

**Figure 3:**
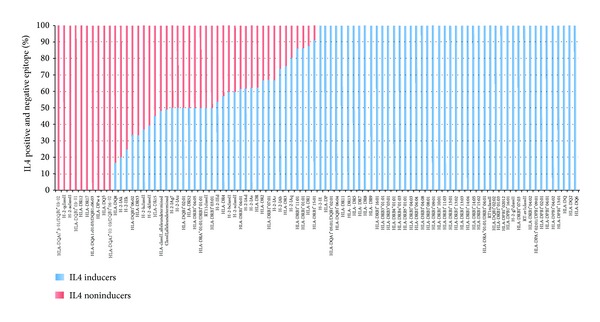
Representing the percentage bar plot of IL4 positive and IL4 negative epitope with corresponding MHC alleles.

We have analyzed the role of MHC alleles to skew the immune response to induce IL4 cytokine. The dataset that comprises 1759 epitopes was derived from 2845 IL4 assays and MHC alleles were undetermined in 1901 (66.8%) assays. The rest of assays (33.2%) were restricted to 91 MHC alleles. Out of these 91 alleles, 34 alleles were observed in IL4 positive as well as IL4 negative assays (Figure 2S). We have also found 10 alleles for which IL4 assays were returned as exclusively negative and 47 alleles for which all the IL4 assays were resulted as exclusively positive (Figure 3). The most promiscuous MHC allele for exclusive IL4 positive assays was HLA-DR7, which could bind to 9 different epitopes and induce IL4 cytokine.

### 3.4. Positional Preference of Residues

**Figure 4:**
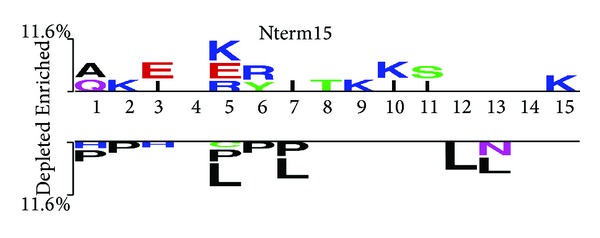
Two-sample logo displaying the positional conservation of amino acid for N15 residue among positive and negative dataset.

The amino acid compositional analysis described the overall dominant residues in IL4 inducing and noninducing peptides. However, this information does not specify the positional preference of specific amino acid residues at specific positions. In order to know the preference of a particular amino acid at different positions or at N- or C-terminals, we created the two-sample logo for our positive and negative IL4 peptides. Two-sample logo as depicted in Figure 4 showed that certain residues are preferred at specific positions; in IL4 inducers charged residues are preferred at 2nd, 5th, 9th, 10th, and 15th positions while leucine or proline residues are abundant in non-IL4-inducing at 1st, 2nd, 5th, 6th, 7th, 12th, and 13th positions. These results clearly suggest that the IL4 inducing and IL4 noninducing MHC class II binders can be discriminated on the basis of residues preferences.

In order to look at position specific proclivity of different PCPs as mentioned in the method section, we calculated the average of every PCP, at every position of N′ and C′ terminal as mentioned in the method section. For every PCP, we found various positions showing discriminating values in plot (Figure 1S); for example, 1, 2, 3, 5, and 12 positions of N′ terminal show high hydrophilicity in IL4+ peptides. Based on these observations we selected discriminating residues for PCP as mentioned in Table 1S. We selected some of the discriminating PCP and looked at the average of the sum of PCP of IL4+, IL4−, IL4 + Nt, IL4 − Nt, IL4 + Ct, and IL − Ct amino acid sequences (Figure 3S) (see the Method section). We found hydrophilicity, pI, amphipathicity, steric, and charge properties, discriminating between IL4+ and IL4− data. On the other hand, the sum of PCP of selected residue positions (see the Method section) showed a significant difference in all the PCPs (Figure 4S).

### 3.5. Motif Search

We next tried to determine exclusive motifs or patterns in IL4 inducing peptides by using MERCI software. We used three types of classification, that is, none, Koolman-Rohm, and Betts-Russell, to determine 100 motifs in peptides. It was observed that Betts-Russell classification significantly discriminated 205 IL4 inducers from noninducers and Koolman-Rohm was significantly distinguished 150 non-IL4-inducers from IL4 inducers (Table 1).

Collectively, motifs generated from all types of classification discriminated 333 positive and 237 negative peptides. The best motifs generated from each classification are listed in Table 2. The most recurring motif in 51 positive peptides was “[hydrophobic]K[hydrophobic] [small][polar]-P[charged].” Similarly, “[aliphatic][hydrophobic][aliphatic][hydrophobic] [aliphatic]-L-[aliphatic]” motif was repeated in 41 IL4 noninducing sequences. Both of these motifs were found to be absent from the alternative datasets.

### 3.6. SVM Based Prediction Model

We developed prediction models using SVM that is widely used in the past for classification models [27–30]. In the present work, we first developed a SVM based model using amino acid and dipeptide composition of the IL4 inducers and noninducers. With this model, we attained a maximum MCC of 0.29 and 0.31, respectively (Table 3). As we have initially observed that the length of the sequence is not contributing to the IL4 inducing or noninducing potential of the peptide, we also developed a SVM model based on amino acid composition, dipeptide composition with length of the peptide, and observed no significant improvement in the performance (Table 2S).

We further developed another SVM model based on binary profile of amino acids of the peptides, where a vector of dimension 20 represents each residue. The compositional variation plot for each residue in IL4 inducing and noninducing peptides and the performance of SVM model based on binary profile of N-/C-terminal residues were also analyzed (depicted in Table 3S).

### 3.7. Hybrid Prediction Model

We adopted a hybrid approach for prediction of IL4 peptides by combining the prediction based on SVM model and motif search. The datasets were first sorted based on the exclusive motifs searched in positive and negative peptides using MERCI. The initial search identified 333 IL4 inducing and 237 IL4 noninducing MHC class II binding peptides, and these sequences were given weightage by adding +1 and −1, respectively, in SVM score in the hybrid method. Additionally, in this hybrid approach, we developed four different models using different input features each time (Table 4). This technique resulted in better performance of each of the four hybrid models over motif or SVM model alone. In the hybrid model, while combining amino acid pairs and motif search, we obtained a maximum MCC of 0.51. Furthermore, fivefold cross-validation technique was used to test the robustness of all prediction models.

### 3.8. Models for Discovering IL4 Inducing Peptides

All the models described above has been developed on main dataset that contain experimentally validated IL4 inducing and noninducing MHC class II binders. These models only can be used for predicting IL4 inducing peptides if users know that their query peptide is MHC class II binders. In order to provide service to the community we developed models on alternate dataset that can be used to discover IL4 peptides in proteins/antigens. As described in Materials and the Methods section our alternate dataset contains negative set/examples random peptide. We developed models on alternate dataset and achieved maximum accuracy of 70% (Table 5).

### 3.9. Model Validation

Performance on independent dataset is one of the best ways to validate a prediction model. As the IEDB database is continuously updated, we extracted the 71-peptide novel entries (deposited after extraction of our dataset) from IEDB for MHC class II binders for which IL4 assay was positive. Out of 71 peptides our best model correctly predicted 49 epitopes at default threshold.

## 4. Discussion

With the advent of the next generation sequencing techniques, the designing of rational vaccines based on the immunogenic features of peptides has become a need of the modern era. Identification of peptides or antigenic that can activate all arms of the immune system is important for designing effective immunotherapy or epitope/subunit vaccine. It means vaccine candidates (peptide/antigen) should have antigenic regions that can activate both B-cell and T-cell epitopes (MHC Class I or II peptides). The Th2 response is very important in vaccine or immunotherapy design against extracellular pathogen. IL4 is the principal cytokine that directs commitment of T cells to Th2 phenotype [31]. Therefore, in this study an attempt had been made to predict the IL4 inducing MHC class II binders.

We extracted experimentally validated MHC class II binding peptides from IEDB with and without IL4 inducing potential. We initially analyzed both these datasets to select important features that could lay the basis for the IL4 inducing capability of the peptide. It is well documented that the binding of peptides to MHC complex is largely dependent on the length of the peptides [32]; thus we also examine the length of both IL4 inducing and noninducing peptides. We observed that the length of both IL4 inducing and noninducing is in the same range. This is the reason that our peptide composition based SVM models developed with peptide length as an additional feature have not resulted in improvement of the performance (Table 2S). Likewise, the length of the peptide and the conservation of amino acid residues at a specific position also play a crucial role in describing the IL4 inducing properties of peptides.

MHC alleles are well documented in literature for thier capability to skew the immune response [33–35]. Our analysis also supports this notion and we have observed 47 MHC alleles that are shown to induce IL4 cytokine. On comparison of IL4 inducing and noninducing reference sequences, it was observed that charged residues preferentially occupy 2nd, 5th, 9th, 10th, and 15th positions in IL4 inducing sequence, while aliphatic and aromatic residues largely reside at 1st, 2nd, 5th, 6th, 7th, 12th, and 13th positions in IL4 noninducing peptides. It is thoughtful that such a differential preference of amino acids might be responsible for activating different factors for downstream signaling. Here, it is important to mention that these positional preferences could not be related to MHC groove as the information was extracted from the sequential comparison of epitopes.

Distinction of different immune epitopes has already been reported with different PCPs in the past [36]. In our analysis, PCPs like hydrophilicity, amphipathy, charge, pI, and so forth (Figures 3S and 4S) showed difference in IL4+ and IL4− peptides both in full length and N/C′ terminal residues. The study with selected residues showed that hydrophilicity, pI, amphipathicity, steric, and charge properties are more profound in IL4+ peptides (Table 1S). We, next, analyzed the IL4 inducing and noninducing reference sequences for the presence of exclusive motifs that may distinguish both these types of sequences, by using MERCI software. Using MERCI, the exclusive motifs could be hunted by employing different classification of amino acids as proposed in the literature. We analyzed our reference IL4 inducing and noninducing dataset on these classifications and found that best results were obtained with Betts-Russell classification. The top ten motifs from each classification, based on the uniqueness in their sequence coverage, were capable of distinguishing 333 IL4 inducing epitopes and 237 non-IL4-inducing epitopes.

Next, we tried to discriminate IL4 inducers and noninducers by use of machine learning technique. For analysis of positional feature of a sequence by SVM, binary patterns of the sequences were used as input. Since binary patterns of peptides could only be applied at a fixed length, we generated different binary inputs by varying the length of amino acids from 9 to 15 through both N and C terminals of a peptide. The performance of SVM model based on these inputs showed a MCC of 0.18. Further, we analyzed the residue and dipeptide compositional vector of IL4 inducer and noninducer sequences. It was observed that the models based on compositional profile performed better than models trained on binary patterns, possibly because this attribute of the sequence does not depend upon the length of the peptide as it has a fixed feature input of 20 and 400 for residue composition and dipeptide composition vector, respectively.

We have also developed hybrid model, by employing the information from motif and machine learning. In the hybrid approach, the weightage has been given to sequences that could be predicted with exclusive motifs searched using MERCI. We observed that the performance was improved up to a value of MCC 0.51 using hybrid of MERCI and amino acid pair. This could be attributed to the role of propensity and exclusive motifs in prediction of IL4 inducing epitopes as it was also publicized for B-cell epitopes [23]. The AAP feature may have some biasness as they incorporate weightage information from the whole data.

Performance of our model on independent dataset was that 69% (49 out of 71) is satisfactory. This performance is comparable with 78.76% sensitivity at fivefold cross-validation on training dataset. In summary, we have developed the *in silico* prediction method that can aid in understanding the IL4 inducing potential of the antigens in computer aided rational vaccine design for better control of diseases.

## 5. Conclusion

The tendency of an epitope to induce IL4 and skew the immune response towards Th2 makes it of great significance in immunotherapy and vaccine designing. Although the induction of IL4 response is a very complex issue that depends on a number of factors like cytokine milieu, MHC haplotype, the costimulatory molecules, and peptide itself, a peptide is an important factor that could be controlled easily while designing a vaccine or immunotherapy. However, Th2 response includes other cytokines like IL5 and IL10; we only focused on IL4 cytokine. Although the experimental evidence for Th2 peptides is limited, our computational analysis appears to support their existence.

Keeping this limitation in mind, we have made an attempt to predict the peptides that may induce IL4 response. In this study we evaluate performance of our models using fivefold cross-validation as well as evaluating performance of our method on an independent dataset. It was observed that our model predicts IL4 inducing peptides with reasonable accuracy. In order to facilitate the scientific community working in the area of subunit vaccine, we have used the above models for developing a webserver IL4pred (http://crdd.osdd.net/raghava/il4pred/).

## Supplementary Material

We are providing three tables and four figures as supplementary document. In these three tables, we are providing the performance of SVM on our main dataset using features like physicochemical properties, compositional feature combined with length of peptide and binary profile of different window length. The figures in supplementary document represent the analysis of physiochemical properties and MHC alleles in our main dataset.

## Figures and Tables

**Table 1:** Exclusive motifs of different class found in IL4 inducing and non-inducing peptides. These motifs were discovered using MERCI software.

Serial no.	Class of motifs	No. of exclusive IL4 inducing peptide	No. of exclusive non-IL4-inducing peptide
1	None	103	137
2	Koolman-Rohm	167	150
3	Betts-Russell	205	128
4	Total unique	**333**	**237**

**Table 2:** Frequency of best motifs discovered using MERCI software in IL4 inducers and IL4 noninducers.

Class of motifs	Found in IL4 inducers	Frequency	Found in IL4 noninducers	Frequency
None	I-N-KI	28	P-D-D-P	22
Koolman-Rohm	[acidic][aliphatic]-K-[aromatic][neutral]-K	31	L[aliphatic][aliphatic]-L [aliphatic]-L[aliphatic]	29
Betts-Russell	[hydrophobic]K[hydrophobic][small][polar]-P[charged]	51	[aliphatic][hydrophobic][aliphatic][hydrophobic] [aliphatic]-L-[aliphatic]	41

Negative sign (−) represents the gaps with the length of 1–5 residues at that position.

**Table 3:** The performances of SVM models developed using various compositional features of peptides on rbf-kernel. The optimized parameters have been given in the brackets.

Thres.	AAC (*g*: 0.001; *c*: 3; *j*: 1)				DPC (*g*: 0.001; *c*: 3; *j*: 1)				AAP (*g*: 0.1; *c*: 1; *j*: 1)			
	Sen.	Spec.	Acc.	MCC.	Sen.	Spec.	Acc.	MCC.	Sen.	Spec.	Acc.	MCC.
−1	97.23	14.56	59.96	0.22	98.67	8.22	57.9	0.17	99.78	0.81	55.16	0.04
−0.9	96.57	18.06	61.18	0.24	97.68	12.26	59.17	0.2	99	4.85	56.56	0.12
−0.8	95.46	20.75	61.79	0.25	97.01	17.25	61.06	0.24	98.89	7.01	57.47	0.15
−0.7	94.03	24.39	62.64	0.26	95.69	21.7	62.33	0.26	98.56	9.7	58.51	0.19
−0.6	92.37	27.36	63.06	0.26	94.47	25.88	63.55	0.29	97.9	12.94	59.6	0.21
−0.5	90.49	30.59	63.49	0.27	92.26	28.71	63.61	0.28	96.46	16.04	60.21	0.22
−0.4	88.38	33.83	63.79	0.27	90.15	33.56	64.64	0.29	95.69	19.68	61.42	0.24
−0.3	85.62	37.2	63.79	0.26	86.17	38.27	64.58	0.28	94.25	23.85	62.52	0.26
−0.2	82.41	41.24	63.85	0.26	83.3	43.53	65.37	0.29	91.92	28.98	63.55	0.27
−0.1	78.87	45.82	63.97	0.26	80.09	48.65	65.92	0.3	88.38	34.23	63.97	0.27
0	75.77	49.6	63.97	0.26	75.44	54.45	65.98	0.31	83.74	42.59	65.19	0.29
0.1	73.12	54.04	64.52	0.28	70.24	59.7	65.49	0.3	77.99	54.85	67.56	0.34
**0.2**	**69.14**	**59.43**	**64.76**	**0.29**	**65.49**	**65.9**	**65.67**	**0.31**	**70.58**	**67.25**	**69.08**	**0.38**
0.3	63.94	63.88	63.91	0.28	60.4	70.62	65.01	0.31	49.12	79.11	62.64	0.29
0.4	58.3	67.79	62.58	0.26	54.76	74.53	63.67	0.3	30.97	88.01	56.68	0.23
0.5	53.1	73.18	62.15	0.27	47.79	78.44	61.6	0.27	21.13	91.64	52.92	0.18
0.6	48.01	76.55	60.87	0.25	39.27	82.35	58.69	0.24	15.27	94.47	50.97	0.16
0.7	40.82	80.19	58.57	0.23	32.08	86.25	56.5	0.21	11.06	96.23	49.45	0.14
0.8	34.85	84.1	57.05	0.21	26.55	89.62	54.98	0.2	7.3	97.84	48.12	0.12
0.9	28.87	87.06	55.1	0.19	19.91	92.18	52.49	0.17	4.65	98.79	47.08	0.1
1	23.45	90.03	53.46	0.18	12.94	95.01	49.94	0.14	2.21	99.46	46.05	0.07

**Table 4:** The performance of hybrid models that combines motif based approach and SVM models developed using various compositional features of peptides on rbf-kernel. The optimized parameters have been given in the brackets.

Thres.	AAC_MOTIF (*g*: 0.01; *c*: 2; *j*: 1)				DPC_MOTIF (*g*: 0.01; *c*: 3; *j*: 1)				AAP_MOTIF (*g*: 0.1; *c*: 1; *j*: 1)			
	Sen.	Spec.	Acc.	MCC.	Sen.	Spec.	Acc.	MCC.	Sen.	Spec.	Acc.	MCC.
−1	99.56	17.92	62.76	0.31	98.89	27.22	66.59	0.39	99.78	20.62	64.09	0.35
−0.9	99.23	25.47	65.98	0.38	97.9	30.05	67.31	0.39	99.12	25.2	65.8	0.37
−0.8	99.23	30.05	68.04	0.42	97.01	33.42	68.35	0.41	99	29.92	67.86	0.41
−0.7	99.12	31.54	68.65	0.43	95.91	37.2	69.44	0.42	98.67	33.69	69.38	0.44
−0.6	98.56	33.29	69.14	0.43	94.69	40.03	70.05	0.42	98.12	36.52	70.35	0.45
−0.5	98.34	34.1	69.38	0.44	93.14	43.26	70.66	0.43	97.01	38.41	70.6	0.45
−0.4	97.9	34.64	69.38	0.43	91.26	45.82	70.78	0.42	96.68	39.62	70.96	0.45
−0.3	97.01	36.12	69.56	0.43	89.16	50.27	71.63	0.43	95.46	42.18	71.45	0.46
−0.2	96.68	37.33	69.93	0.43	86.17	56.33	72.72	0.45	93.58	45.15	71.75	0.45
−0.1	94.58	39.62	69.81	0.42	83.19	60.92	73.15	0.46	91.04	47.98	71.63	0.44
0	91.26	45.28	70.53	0.42	79.09	64.96	72.72	0.45	88.16	54.45	72.96	0.46
**0.1**	**71.13**	**71.56**	**71.32**	**0.43**	**76.33**	**69.41**	**73.21**	**0.46**	84.07	63.21	74.67	0.49
**0.2**	53.43	88.95	69.44	0.44	72.23	72.51	72.36	0.45	**78.76**	**72.1**	**75.76**	**0.51**
0.3	47.9	93.8	68.59	0.46	67.37	76.28	71.39	0.43	66.26	81.13	72.96	0.47
0.4	43.92	95.69	67.25	0.45	63.83	78.57	70.47	0.42	54.98	89.08	70.35	0.46
0.5	41.15	97.84	66.71	0.46	58.63	83.02	69.62	0.42	48.34	92.18	68.1	0.44
0.6	39.93	98.52	66.34	0.46	53.43	86.25	68.23	0.41	44.47	95.01	67.25	0.44
0.7	38.72	99.19	65.98	0.46	49.12	89.49	67.31	0.41	41.48	96.5	66.28	0.44
0.8	37.17	99.46	65.25	0.45	44.47	91.91	65.86	0.4	38.61	97.84	65.31	0.44
0.9	36.06	99.46	64.64	0.44	40.04	93.8	64.28	0.39	35.62	98.79	64.09	0.43
1	34.85	99.6	64.03	0.43	35.62	95.69	62.7	0.38	32.85	99.46	62.88	0.42

**Table 5:** The performance of SVM models developed on alternate dataset using various compositional features of peptides on rbf-kernel.

Features	Param.	Threshold	Sensitivity	Specificity	Accuracy	MCC	ROC
AAC	*g*: 0.001; *c*: 4; *j*: 1	0	65.48	64.37	64.92	0.3	0.68
DPC	*g*: 0.001; *c*: 9; *j*: 5	0	68.22	67.61	67.92	0.36	0.75
AAP	*g*: 0.05; *c*: 3; *j*: 1	0.1	69.44	72.28	70.86	0.42	0.78
