# Supplementary material for: Prediction of IL4 Inducing Peptides
Source: Clin Dev Immunol. 2013 Dec 30;2013:263952. doi: 10.1155/2013/263952 (PMC3893860; doi:10.1155/2013/263952)
Supplement: Supplementary file 1 — We are providing three tables and four figures as supplementary document. In these three tables, we are providing the performance of SVM on our main dataset using features like physicochemical properties, compositional feature combined with length of peptide and binary profile of different window length. The figures in supplementary document represent the analysis of physiochemical properties and MHC alleles in our main dataset. [file 263952.f1.pdf]

Table-1S: This table presents SVM based prediction results for different physiochemical properties, which show difference between Il4-positive and IL4-negative peptides, at various positions on amino acid sequence.

Input	Method	Residue position	Param	Thres	Sen	Spec	Acc	MCC	ROC
**C-ter**	Charge	3,4,5,6,9,10,12,15	g:0.01 c:15 j:1	0.2	55.7	55.85	55.77	0.12	0.57
	Hydrophobicity	1,2,3,5,11,12,13,14,15	g:0.01 c:6 j:1	0.2	57.78	50.18	54.17	0.08	0.54
	pI	3,4,5,6,9,10,12,14	g:0.01 c:1 j:1	0.1	56.18	53.72	55.01	0.1	0.54
	Steric effect	5,6,8,9,11,12,13	g:0.0005 c:15 j:1	0.9	58.75	51.24	55.18	0.1	0.55
	Hybrid		g:0.005 c:5 j:2	0.7	58.27	52.3	55.43	0.11	0.57
**N-ter**	Charge	5,6,7,8,9,10,15	g:0.5 c:1 j:1	0	60.83	57.8	59.39	0.19	0.6
	Hydrophilicity	1,2,3,5,12	g:0.1 c:7 j:1	-0.2	63.72	55.5	59.81	0.19	0.6
	Hydropathy	1,2,3,5,6	g:0.01 c:4 j:1	0.2	58.75	53.55	56.28	0.12	0.58
	Amphipathy	1,2,3,5,6,12	g:0.01 c:7 j:1	0.1	54.57	62.77	58.47	0.17	0.6
	pI	3,4,5,6,9,10,12,14	g:0.005 c:1 j:1	-0.1	58.75	57.27	58.05	0.16	0.59
	Hybrid		g:0.01 c:1 j:1	0.1	60.67	58.69	59.73	0.19	0.63
**Hybrid**	Hybrid C’+N’		g:0.01 c:1 j:1	0.1	62.44	56.38	59.56	0.19	0.63

Table 2S: Performances of various amino acid sequence based features with Support Vector Machine classifier on processed data (8-22 residue length) by incorporating length feature with other features. The best kernel optimized for all the results is rbf-kernel.

**Method**	**Param**	**Thres**	**Sen**	**Spec**	**Acc**	**MCC**	**ROC**
**AA Composition + Length**	g:0.005 c:1 j:1	0.2	65.15	65.9	65.49	0.31	0.7
**DP Composition +Length**	g:0.001 c:1 j:2	0.5	68.47	62.26	65.67	0.31	0.7
**AAP + Length**	g:0.1 c:1 j:1	0.2	71.57	64.02	68.10	0.36	0.72
**MERCI_AAC + Length**	g:0.005 c:1 j:1	0.2	73.45	71.29	72.48	0.45	0.82
**MERCI_DPC + Length**	g:0.005 c:1 j:1	0.2	69.03	78.57	73.33	0.47	0.84
**MERCI_AAP + Length**	g:0.05 c:1 j:1	0.2	78.32	71.56	75.27	0.5	0.84

Table 3S: The performance of various features with SVM light on 5 folds cross validation

Method	Param	Pos*	Neg*	Thrs	Sen	Spec	Acc	MCC	ROC
**Binary -9aa Cter**	g:0.05 c:7 j:4	893	738	0.2	51.4	60.84	55.67	0.12	0.58
**Binary -10aa Cter**	g:0.05 c:15 j:5	871	727	0.1	57.29	54.06	55.82	0.11	0.57
**Binary -11aa Cter**	g:0.1 c:3 j:4	833	668	0.1	58.58	51.2	55.3	0.1	0.56
**Binary -12aa Cter**	g:0.1 c:9 j:2	811	656	0.1	59.43	54.57	57.26	0.14	0.57
**Binary -13aa Cter**	g:0.1 c:1 j:4	704	632	0.3	53.41	55.22	54.27	0.09	0.56
**Binary -14aa Cter**	g:0.1 c:1 j:2	666	610	0.2	60.96	51.97	56.66	0.13	0.59
**Binary -15aa Cter**	g:0.01 c:17 j:2	623	564	0.3	60.03	51.6	56.02	0.12	0.56
**AAC -9aa Cter**	g:0.001 c:6 j:3	893	738	0.2	62.37	53.93	58.55	0.16	0.6
**AAC -10aa Cter**	g:0.0005 c:2 j:1	871	727	0.3	63.15	56.12	59.95	0.19	0.62
**AAC -11aa Cter**	g:0.001 c:1 j:1	833	668	0.3	65.91	58.38	62.56	0.24	0.66
**AAC -12aa Cter**	g:0.005 c:1 j:1	811	656	0.2	61.53	65.24	63.19	0.27	0.67
**AAC -13aa Cter**	g:0.001 c:4 j:1	704	632	0.2	63.35	62.5	62.95	0.26	0.66
**AAC -14aa Cter**	g:0.0005 c:4 j:1	666	610	0.2	63.51	58.85	61.29	0.22	0.65
**AAC -15aa Cter**	g:0.001 c:6 j:1	623	564	0.1	65.81	61.52	63.77	0.27	0.68
**DPC -9aa Cter**	g:0.001 c:2 j:1	893	738	0.1	62.71	61.11	61.99	0.24	0.67
**DPC -10aa Cter**	g:0.001 c:2 j:2	871	727	0.1	64.64	58.05	61.64	0.23	0.66
**DPC -11aa Cter**	g:0.001 c:6 j:1	833	668	0.1	63.51	58.38	61.23	0.22	0.67
**DPC -12aa Cter**	g:0.005 c:15 j:1	811	656	0.1	66.34	61.74	64.28	0.28	0.69
**DPC -13aa Cter**	g:0.001 c:5 j:1	704	632	0	68.89	59.18	64.3	0.28	0.69
**DPC -14aa Cter**	g:0.001 c:1 j:1	666	610	0.1	66.82	56.89	62.07	0.24	0.66
**DPC -15aa Cter**	g:0.001 c:4 j:3	623	564	0.1	64.04	63.83	63.94	0.28	0.68
**Binary -9aa Nter**	g:0.05 c:7 j:1	893	738	0.1	57.45	53.93	55.86	0.11	0.58
**Binary -10aa Nter**	g:0.1 c:2 j:3	871	727	0.1	61.19	51.44	56.76	0.13	0.6
**Binary -11aa Nter**	g:0.01 c:8 j:1	833	668	0.2	59.18	54.19	56.96	0.13	0.58
**Binary -12aa Nter**	g:0.01 c:10 j:1	811	656	0.2	58.2	59.91	58.96	0.18	0.59
**Binary -13aa Nter**	g:0.1 c:1 j:1	704	632	0.1	56.96	58.86	57.86	0.16	0.62
**Binary -14aa Nter**	g:0.1 c:1 j:1	666	610	0.1	55.56	62.13	58.7	0.18	0.61
**Binary -15aa Nter**	g:0.005 c:2 j:1	623	564	0.2	59.71	54.43	57.2	0.14	0.6
**AAC -9aa Nter**	g:0.005 c:8 j:3	893	738	0.1	62.37	64.63	63.4	0.27	0.68
**AAC -10aa Nter**	g:0.001 c:2 j:1	871	727	0.2	65.33	57.63	61.83	0.23	0.65
**AAC -11aa Nter**	g:0.005 c:1 j:2	833	668	0.2	65.19	67.66	66.29	0.33	0.71
**AAC -12aa Nter**	g:0.005 c:1 j:2	811	656	0.2	65.1	67.23	66.05	0.32	0.72
**AAC -13aa Nter**	g:0.01 c:1 j:2	704	632	0.1	64.77	67.25	65.94	0.32	0.71
**AAC -14aa Nter**	g:0.005 c:2 j:1	666	610	0	69.97	60.82	65.6	0.31	0.71
**AAC -15aa Nter**	g:0.001 c:20 j:2	623	564	0.1	69.34	63.83	66.72	0.33	0.7
**DPC -9aa Nter**	g:0.001 c:6 j:5	893	738	0.1	65.06	60.98	63.21	0.26	0.68
**DPC -10aa Nter**	g:0.001 c:2 j:1	871	727	0.1	63.61	63.82	63.7	0.27	0.69
**DPC -11aa Nter**	g:0.001 c:1 j:1	833	668	0.2	67.95	60.03	64.42	0.28	0.69
**DPC -12aa Nter**	g:0.005 c:15 j:2	811	656	0.1	71.15	61.28	66.73	0.33	0.73

**DPC-13aa Nter**	g:0.001 c:2 j:2	704	632	0.2	62.64	67.88	65.12	0.3	0.71
**DPC-14aa Nter**	g:0.001 c:9 j:1	666	610	0.1	61.26	69.02	64.97	0.3	0.7
**DPC-15aa Nter**	g:0.001 c:6 j:1	623	564	0	68.38	59.93	64.36	0.28	0.69

* Variation in the number of positive and negative dataset on window length is because we have considered only those epitopes that have length above or equal to that particular window length.

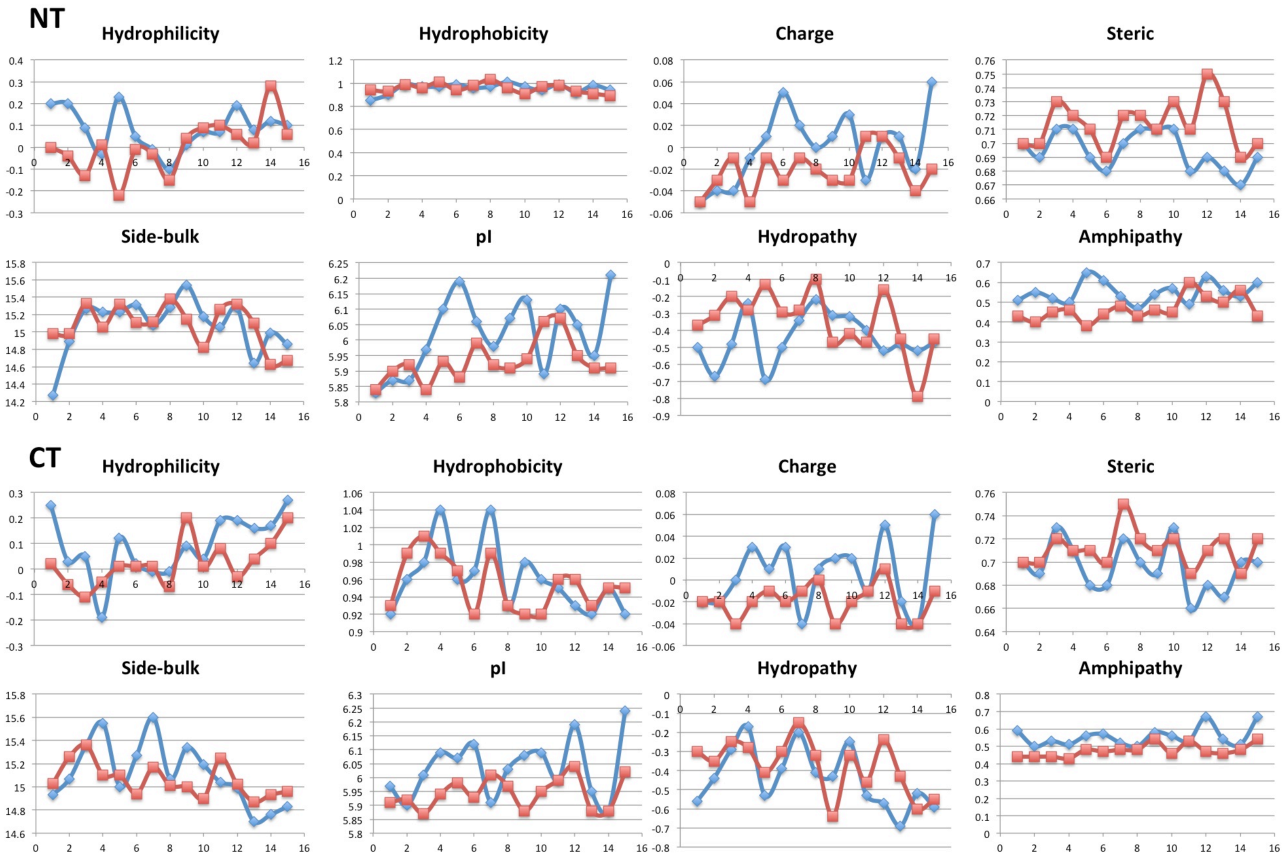

Figure-1S: showing position specific distribution of different physiochemical properties at C-terminal and N-terminal. NT and CT denotes for N-terminal and C-terminal respectively. Blue color line represent IL4+ and red color line denotes IL4- data.

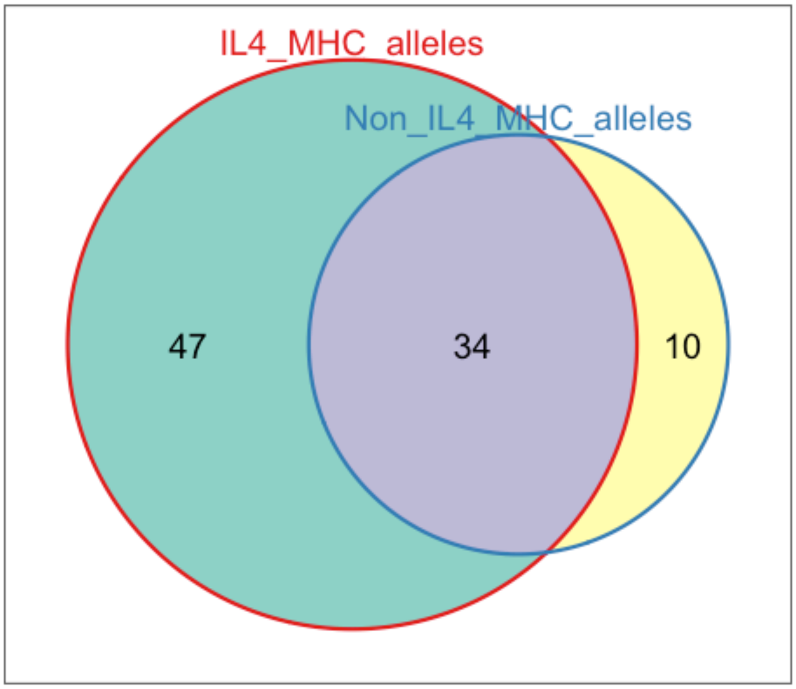

Figure-2S: The Venn Diagram to represent the distribution of MHC alleles among IL4 assays.

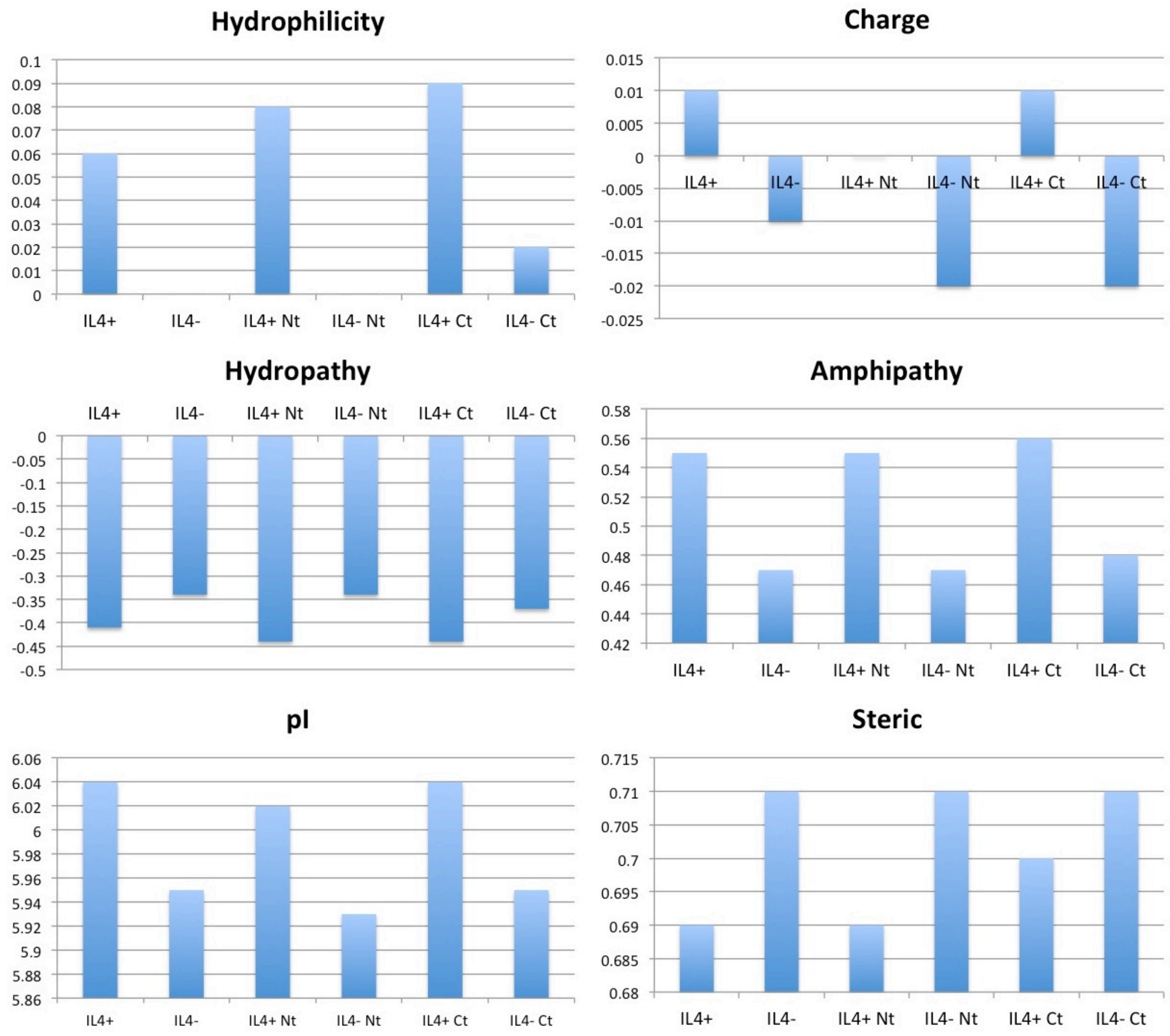

Figure-3S: showing preference of different physiochemical properties between IL4-positive and IL4-negative peptides. Here IL4+, IL4-, Nt and Ct denotes for IL4-positive, IL4-negative, N-terminal and C-terminal respectively.

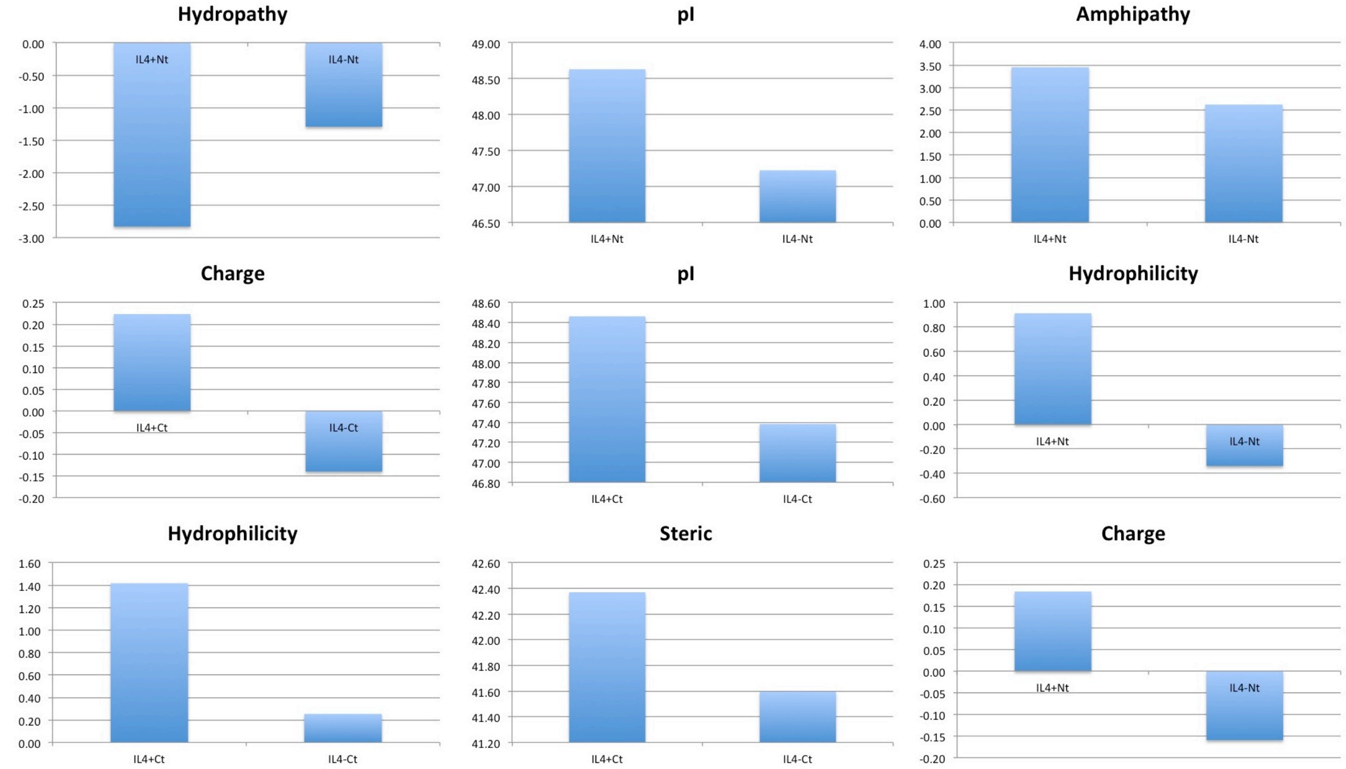

Figure-4S: showing preference of different physiochemical properties between IL4-positive and IL4-negative peptides. Here Y-axis shows cumulative sum of that property for selected residues (dominating in IL4-positive) from figure 1S. Here IL4+, IL4-, Nt and Ct denotes for IL4-positive, IL4-negative, N-terminal and C-terminal respectively.
